# Are Extracorporeal Shock Waves Just a Therapeutic Tool?

**DOI:** 10.3390/diagnostics14212440

**Published:** 2024-10-31

**Authors:** Bernardo Gialanella, Laura Comini, Gian Pietro Bonometti, Fabio Vanoglio, Andrea Bettinsoli, Raffaele Santoro, Adriana Olivares, Alberto Luisa

**Affiliations:** 1 Istituti Clinici Scientifici Maugeri IRCCS, Neuromotor Rehabilitation Unit, Institute of Lumezzane, 25065 Lumezzane, Italy; gialanellab@gmail.com (B.G.); andrea.bettinsoli@icsmaugeri.it (A.B.); giampietro.bonometti@icsmaugeri.it (G.P.B.); fabio.vanoglio@icsmaugeri.it (F.V.); raffaele.santoro@icsmaugeri.it (R.S.); alberto.luisa@icsmaugeri.it (A.L.); 2 Istituti Clinici Scientifici Maugeri IRCCS, Scientific Direction, Institute of Lumezzane, 25065 Lumezzane, Italy; adriana.olivares@icsmaugeri.it

**Keywords:** ESWT, evaluation, pain threshold, site of pain, musculoskeletal diseases

## Abstract

Background: Focused extracorporeal shock wave therapy (ESWT) has been successfully used to treat musculoskeletal conditions, but ESWT stimulates nociceptors, causing pain deep in the tissue during treatment. The occurrence of pain during ESWT is a side effect, but it can help identify painful sites and assess minimum (MiTI) and maximum (MaTI) pain thresholds to ESWT pressure stimuli. This topic has received limited attention in literature. Methods: This observational study describes a specific approach to using ESWT to study pain in 71 patients. The approach proposes moving the ESWT transducer head of the device over the entire joint surface, progressively increasing the energy level until the patient experiences pain. Results: In the study, MiTI and MaTI were 0.218 ± 0.090 and 0.416 ± 0.165 mJ/mm^2^ in the affected joint and 0.282 ± 0.128 and 0.501 ± 0.174 mJ/mm^2^ in the contralateral homologous healthy joint, being significantly lower in the affected joint (MiTI: *p* < 0.001 and MaTI: *p* = 0.003, respectively). ESWT induced pain in 94.37% of the sites with the highest subjective pain and in a greater number of sites (204) than digital pressure (123) (*p* < 0.001). All sites with digital pressure pain also had ESWT pain. Conclusions: These results suggest that the ESWT device may be useful in investigating pain in musculoskeletal conditions and tailoring therapy.

## 1. Introduction

Extracorporeal shock wave therapy is a remarkable non-invasive treatment option for various musculoskeletal conditions, demonstrating effectiveness in reducing pain and improving functional outcomes.

The first studies into the potential medical applications of this technique date back to the early 1970s, when shock waves were used to break up kidney crystalline aggregates. From the early 1980s and for about a decade, the use of shock waves was limited to urology.

It was only at the beginning of the 1990s that the technique was redirected towards pathological neo calcifications in musculoskeletal disorders, and the first results of the use of this treatment in bone and musculoskeletal pathologies became known. Already, the first results of the use of this treatment in bone and musculoskeletal pathologies have led to the indication of this treatment also for musculoskeletal disorders [1].

Shock waves are defined as pressure waves that propagate in three dimensions and typically induce a significant increase in pressure within a few nanoseconds [2]. They are characterized by very rapidly rising positive pressure pulses from 5 to 120 MPa in about 5 ns, followed by a decline to negative pressure values of −20 MPa [3].

The positive and negative phases of shock waves have specific effects on the interfaces between different tissues and their different densities. In the positive phase, high pressure shock waves may hit an interface and be reflected, or they may pass through and be gradually absorbed. During the negative phase, the shock wave creates cavitation at the tissue interfaces, resulting in the subsequent formation of air bubbles. The air bubbles then implode at high speed, creating a second wave of shock waves or micro-jets of fluid (jet stream) [4].

These forces can stimulate the tissue repair process. Chao et al. [5]. observed that shockwave treatment of rat Achilles tendon tenocytes induced upregulation of proliferating cell nuclear antigen (PCNA), collagen types I and III, and transforming growth factor-beta1 (TGF-beta1) gene expression, followed by increases in nitric oxide (NO) production, TGF-beta1 release, and collagen synthesis.

Nitric oxide and vascular endothelial growth factor (VEGF) have been shown to be important mediators of angiogenesis. Wang et al. [6] showed that shock wave therapy induced neovascularization and tissue proliferation associated with early release of angiogenesis-related factors including endothelial nitric oxide synthase (eNOS) and vascular endothelial growth factor (VEGF) at the tendon-bone junction in rabbits.

As a result of these effects, the use of shockwave therapy has been extended to a variety of musculoskeletal conditions, with non-unions and tendinopathies being by far the largest group of indications [7].

Shock wave energy flows through an area with a perpendicular orientation to the direction of propagation (and its unit is mJ/mm^2^). On the basis of the energy flux density, Rompe et al. [8] classified the shock wave treatment as low (<0.08 mJ/mm^2^), medium (<0.28 mJ/mm^2^), and high (<0.60 mJ/mm^2^). Usually, the energy flux density used in clinical practice ranges from 0.001–0.4 mJ/mm^2^. At low and medium energy flux density, NO is released, and its analgesic, angiogenic, and anti-inflammatory effects are observed, being very useful in clinical treatment [2]; at high energy flux density, fragmentation and destruction of solid bodies can be induced [9].

The shock waves work through a complex interplay of biological mechanisms.

The shock waves induce controlled mechanical stress to the tissues, resulting in micro-trauma and activation of the body’s repair mechanisms. This mechanical stimulation increases the synthesis and organization of collagen fibers, which are essential for tendon and ligament repair [10].

The pain-relieving effects of shock waves are partly attributed to their impact on pain pathways. Shock waves can modulate the release of pain mediators, such as prostaglandins and cytokines, and influence neural mechanisms involved in pain perception. In addition, shock waves can activate endogenous pain control systems, including the release of endorphins and activation of descending inhibitory pathways [10].

Shock waves increase local blood flow and promote angiogenesis, both crucial for tissue healing. Mechanical stimulation increases the expression of angiogenic factors such as vascular endothelial growth factor and fibroblast growth factor, leading to the formation of new blood vessels. This improved microcirculation increases the delivery of oxygen and nutrients to the damaged tissue, facilitating repair and regeneration [10,11].

The stimulation of collagen production, modulation of pain pathways, and improvement of microcirculation collectively contribute to promote tissue repair and reduce discomfort in various musculoskeletal conditions.

Basically, there are two types of shock waves that can be generated and applied to human tendons: radial shock wave therapy and focused shock wave therapy.

In clinical practice, the focused shock wave is extensively used; it can reach deeper into tissues and concentrate the energy flow in smaller areas compared to radial waves [12].

Because of biological effects, shock waves have been successfully used to treat a variety of musculoskeletal conditions, including pseudoarthrosis, delayed fracture healing, bone marrow oedema and early osteonecrosis, insertional tendinopathies such as plantar fasciitis and Achilles tendon fasciitis, calcific tendinitis of the rotator cuff, and tennis elbow [13,14,15].

Recent systematic reviews and meta-analyses show that shock wave therapy is an effective treatment for plantar fasciitis, with success rates ranging from 50% to 94%, depending on operator experience and the shockwave device used [16].

However, shock waves excite nociceptors, causing pain deep in the tissue during treatment [17,18,19]. Shock wave-induced pain occurs earlier in the injured tissue during treatment [19,20] and increases with increasing energy flux density [18]. The pain induced is influenced by the type of shock wave [13,21] and may be a reason for patients to discontinue therapy [13].

The occurrence of pain during shock wave therapy is a side effect. However, the ability to elicit pain with stimuli of known characteristics may allow the shock wave device to evaluate the “minimum threshold of intensity” (MiTI) and the “maximum threshold of intensity” (MaTI). MiTI and MaTI are important parameters for assessing pain in patients with musculoskeletal diseases [22,23].

The MiTI and MaTI levels are lower in inflamed and damaged tissue [24,25] and increase after therapy [26,27,28] and can provide information on the degree of tissue damage and disease progression [26,27,28].

Despite this, only a few studies have measured MiTI and MaTI with shock wave devices [20,26], and they did not clarify whether the shock wave device can provide information that allows the assessment of patients with musculoskeletal pain. For this reason, the device is currently used only for the treatment of musculoskeletal diseases.

The aim of the study is to investigate whether a focused extracorporeal shockwave therapy (ESWT) device can provide information for the assessment of pain in patients with musculoskeletal diseases.

## 2. Methods

The study was conducted between April and December 2023 in accordance with the principles of the Declaration of Helsinki. The study protocol was approved by the Technical and Scientific Committee of our Institute and the Maugeri Ethics Committee (CE 2726).

The study used a focused ESWT device, the Piezo Wave 2 in TPST mode from the Richard WOLF company (Knittlingen, Germany), to deliver low-intensity focused shock waves. The device is equipped with knobs to program the frequency (range 1–8 Hz) and energy density (1–20 energy levels corresponding to 0.092–0.822 mJ/mm^2^, respectively) of the shock waves as well as a transducer head for transmitting the waves to the patient. Additionally, the device uses interchangeable gel pads that, when placed on the transducer head, allow the desired depth of penetration to be achieved.

### 2.1. Study Sample

The study was conducted on a sample of patients who were admitted to our Rehabilitation Institute for ESWT treatment of unilateral musculoskeletal pain in the shoulder, elbow, hip, knee, and foot caused by insertional tendinopathies and calcific tendinitis of the rotator cuff and tennis elbow, greater trochanteric pain syndrome, patellar tendinopathy, Achilles tendinitis, heel bursitis, and plantar fasciitis.

Patients with coagulation disorders or those who used anticoagulants, patients with pacemakers, pregnant women, those with local infections, cancer patients, patients with cognitive deficits (such as Alzheimer’s disease or senile dementia), patients with neurological diseases that could cause pain or make data evaluation and collection difficult, and patients who did not provide informed consent were excluded from the study. Patients were also excluded if they were taking NSAIDs and corticosteroids at the time of enrolment, as these drugs can reduce subjective pain and alter tissue responses, which could affect pain ratings.

Patients admitted to the study were informed that ESWT could cause discomfort and pain and were instructed to notify the physician when pain occurred during the ESWT evaluation and when the pain reached an unacceptable level.

### 2.2. Clinical Evaluation

At the time of enrollment, the patient’s demographical and clinic data were collected, including age, sex, weight, height, comorbidities, use of NSAIDs or corticosteroids, duration of pain (days), and whether the patient had previously undergone ESWT.

Furthermore, the Cumulative Illness Rating Scale (CIRS) [29] and the Roles and Maudsley scale were employed [30] to assess the severity of illness and the impact on activities, respectively. The Cumulative Illness Rating Scale (CIRS) was used at admission to evaluate comorbidities [29]. In this study, the average severity of all comorbidities (severity index) was considered.

The Roles and Maudsley score was used to evaluate pain and activity limitation as classified by four categories (1 point = excellent, 2 points = good, 3 points = fair, and 4 points = poor) [30].

The locations of joint discomfort were also documented. The study considered the sites of the highest subjective joint pain reported by the patient and the site of pain induced by moderate finger pressure, as identified by the physician.

### 2.3. ESWT and Evaluation by Device

The evaluation using the ESWT device was performed on a patient lying on a bed in a position suitable for joint evaluation.

The transducer head, with a 20 mm gel pad attached, was moved in a slow deliberate manner over the entire joint surface by the physician, who made use of both horizontal and vertical movements while maintaining the transducer head in a perpendicular position relative to the surface of the skin.

The device was used at a fixed frequency of 6 Hz for the entire evaluation period, while the energy density level of the device was increased in a step-wise manner from 0.

In particular, the physician conducted a comprehensive evaluation of the entire joint surface by moving the transducer head and initiating the examination with the lowest level of energy intensity (level 1). In the absence of pain at any site of the joint, the physician proceeded with the assessment, incrementally increasing the energy intensity. If there was no pain at this energy level, the physician continued with further assessments, gradually increasing the energy intensity until a sensation of pain appeared.

The physician recorded the intensity that produced the first pain sensation and defined it as the “minimum threshold intensity” (MiTI) and the areas of joint pain detected by ESWT (Table 1). The procedure was continued with a progressive increase in energy intensity until the patient experienced intolerable pain (maximum threshold intensity) (MaTI).

The assessment was performed first on the injured joint and then on the contralateral homologous healthy joint. This allowed physicians to identify pain sites in the injured joint and measure MiTI and MaTI in both the injured and contralateral homologous healthy joints.

MiTI and MaTI were assessed at baseline and 1 and 2 weeks, at the beginning of three different ESWT sessions. An ESWT session (2000 pulses at 6 Hz with tolerable pain intensity) was performed immediately after each pain threshold assessment.

### 2.4. Statistical Analysis

#### Sample Size Determination

Based on preliminary data, for sample size calculation, we assumed a baseline difference of 35% between MiTi in the affected joint compared to the healthy joint; with this assumption, α = 0.05 and β = 0.80, and 27 patients were calculated. In addition, looking at the difference between MiTi over time, we hypothesized a 16% increase in MiTi in the affected joint after 2 weeks of treatment; with α = 0.05 and β = 0.80, and 60 patients were expected. Taking into account the possibility of dropouts, a total sample size of 70 patients was planned for recruitment.

The statistical analysis was conducted using Statistica version 6 (StatSoft, Tulsa, OK, USA; 2001) and included descriptive analyses (number, mean, percentage), chi-square test (Fisher exact or Pearson as appropriate), Student’s *t*-test, and Friedman test for repeated measurements and, in case of statistically significant differences among groups, followed by a post hoc analysis with Dunn’s correction. P was considered statistically significant for value < 0.05.

## 3. Results

The study was conducted in a sample of 71 patients with musculoskeletal pain (Table 2).

Table 3 shows details of the MiTI and MaTI as measured by ESWT.

At baseline, MiTI and MaTI in the affected joints were significantly lower than in the homologous healthy joints (Table 3, *p* < 0.001 and *p* = 0.003, respectively), and the differences in MiTI and MaTI between the affected and healthy joints were 0.063 ± 0.064 and 0.084 ± 0.084 mJ/mm^2^, respectively. At this time, the percentage of patients with low MiTI (energy levels 1–5, corresponding to 0.092–0.0182 mJ/mm^2^) in the affected was 84.50% vs. 19.72% in the healthy joints, and the percentage was 14.10% vs. 64.79% for energy levels 6–10 (0.220–0.351 mJ/mm^2^) and 1.40% vs. 15.49% for energy levels ≥ 11 (≥0.388 mJ/mm^2^) in the affected and healthy joints, respectively. Table 3 also highlights that in hip joints, MiTI and MaTI were higher than in the other single-considered joints.

**Figure 1:**
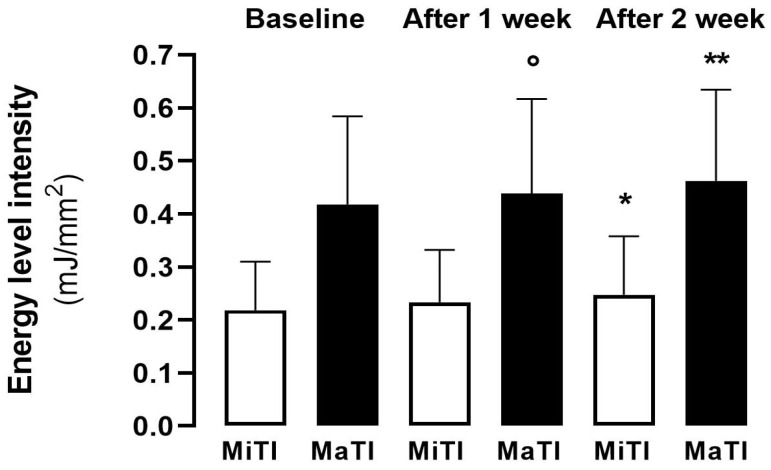
Comparisons of MiTI and MaTI values during the three ESWT sessions in all affected joints. Legend: * for comparison between “After 2 week and Baseline in MiTI” being *p* = 0.001; ° for comparison between “After 1 week and After 2 weeks in MaTI” being *p* = 0.0106; ** for comparison between “After 2 week and Baseline in MaTI” being *p* < 0.0001.

Figure 1 describes the MiTI and MaTI in the affected joints at baseline and at 1 and 2 weeks after the application of the ESWT sessions.

The Friedman test was statistically significant for both MiTI and MaTI comparisons during the three ESWT sessions (both *p* < 0.0001). In the affected joint, both MiTI and MaTI values were significantly higher at 2 weeks compared to baseline (*p* = 0.001 and *p* < 0.0001 respectively, Figure 1), with MaTI values significantly different between the second and third ESWT sessions (*p* = 0.0106, Figure 1). In the healthy joint, no significant differences in thresholds were observed at different times.

In the study sample, the MiTI induced pain in 94.37% of the sites where the patient reported the highest subjective pain. Table 4 details the number of joint sites where pain was elicited by the ESWT and digital pressure.

This table shows that the number of painful sites identified by the ESWT (204 sites, 52.85%) was higher than that identified by the digital pressure (123 sites, 31.90%), and the difference between the two techniques was significant (chi-squared 13.29; *p* < 0.001). The ESWT induced pain in all sites where there was pain induced by digital pressure and in 81 sites where digital pressure pain was absent.

At baseline, MiTI and MaTI of the injured (*p* = 0.005; *p* = 0.004) and healthy (*p* = 0.005; *p* = 0.001) joints were lower in females than in males, while there were no differences between normal weight and overweight patients (*p* = ns) and patients who had experienced ESWT and those who had not (*p* = ns). During ESWT treatment, no patient interrupted the therapeutic ESWT session due to pain caused by the device.

## 4. Discussion

The study aimed to test whether the ESWT device could provide information to assess pain in patients with musculoskeletal diseases. The study found that at baseline, the MiTI scores were lower in the injured joint than in the homologous healthy joint. These data suggest that the ESWT device induces the first sensation of pain in injured tissue at lower energy levels than in healthy tissue. This is because the threshold for mechanical nociceptors is lower in injured tissue than in healthy tissue, and therefore mechanical nociceptors can be excited by lower energy stimuli [17,18]. It is not possible to compare the data of this study with those of other authors because the literature on this subject is scarce and the few studies evaluating MiTI and MaTI with ESWT are not specific [20,26].

However, the results of the study are consistent with previous studies in the literature that have evaluated pain thresholds with pressure algometers and found lower MiTI in injured tissue [24,31,32].

In the injured joint, MiTi was lower than in the homologous healthy joint, and the percentage of patients with low MiTi was very high compared to what was observed in the contralateral joint.

Evaluation of the differences in MiTI between injured and healthy joints can provide information about the severity of tissue damage. In damaged tissue, the threshold of mechanical nociceptors is negatively correlated with tissue damage [24,31,33,34]. This means that the greater the tissue damage, the lower the nociceptor threshold. Therefore, by comparing the MiTI of an injured joint with that of an uninjured joint, the physician can determine the degree of tissue damage. In particular, the detection of large and significant differences in MiTI between injured and healthy joints can lead to the assumption of greater tissue damage. Furthermore, the fact that the MiTI in the healthy joint does not vary over time suggests that its changes in the injured joint are not random but can be attributed to an improvement in tissue damage where there has been treatment with ESWT. The study also assessed patients with musculoskeletal pain after two sessions of ESWT and found that the Roles and Maudsley scores were lower and MiTI and MaTI scores were higher than at baseline. These data are consistent with those of a recent meta-analysis that reported lower pain levels and higher pain thresholds in patients treated with ESWT [26].

The results found in this study may be due to the fact that the therapy had reduced tissue damage, leading to a reduction in subjective pain and an increase in pain thresholds, so that more intense stimuli were required to evoke the first sensation of pain. The increase in thresholds at the end of therapy may therefore indicate that the clinical picture has improved and that the therapy carried out is effective.

The study also used the focused ESWT device to measure MaTI in patients with musculoskeletal pain. The study showed that MaTI values at baseline were lower in the injured joint than in the homologous healthy joint, indicating that the maximum tolerable intensity in injured tissue is lower than in the healthy joint.

Therefore, the MATI measurement can also provide information on the severity of tissue damage. In addition, the MATI can guide the physician in the delivery of ESWT sessions. Systematic research recommends three treatment sessions one-= week apart, with 2000 impulses per session and the highest energy flux density the patient can tolerate [35]. Therefore, by knowing the MaTI, the clinician can deliver a treatment that is not excessively painful and is therapeutic.

In the study, no patient interrupted the ESWT sessions due to pain caused by the device. This may be due to the way the ESWT session was delivered, which required the transducer head of the device to be moved on the joint surface, and the energy intensity was the highest tolerable between MITI and MaTI.

Finally, the focused ESWT device was employed to locate painful joint sites.

The study found that the MiTI induced pain in 94.36% of the sites where the patient reported the greatest subjective pain. This finding shows that there is a high degree of agreement between MiTI-induced pain and subjective pain [36,37] and suggests that the ESWT device, when used according to a specific procedure, can identify painful areas of joints.

This is due to the fact that the threshold for mechanical nociceptors is lower in injured tissue [24,25]; hence, using the ESWT device with the same energy as the MiTI, it is possible to induce pain only in the affected tissue and thus discriminate between healthy and injured tissue.

The study also compared the pain points identified by the focused ESWT device with those identified by digital pressure, which is commonly used in clinical practice to locate and assess the pain [38], and found that the MiTI induced pain in a greater number of joint sites than digital pressure.

This indicates that the focused ESWT device also elicits pain in areas where there is no digital pressure pain. It is therefore more reliable in identifying painful joint areas and, consequently, damaged anatomical structures underlying the painful area.

This is because the focused ESWT device penetrates deep into the tissue and concentrates mechanical energy in deep areas, allowing identification of pain in sites that cannot be reached by digital pressure. Moreover, the focused device concentrates mechanical energy on smaller areas of the joint [39,40], allowing for a more precise discrimination between healthy and damaged tissues than is possible with digital pressure.

Regardless, the results of pain localization suggest that the EWST devices can be used to map the pain sites and thus identify the damaged anatomical structures underlying the pain sites. This can help the physician to confirm an existing diagnosis and guide him toward new pain sites during ESWT treatment sessions, as the flow of energy needs to be focused mainly on the painful areas.

In the study, women had lower MiTI and MaTI values than men in both injured and healthy joints; this difference between genders has also been found in previous studies that measured pain thresholds using a pressure algometer [41,42]. However, the specific mechanisms underlying the observed disparity are not yet clear, and it has been suggested that an interaction of biological, psychological, and socio-cultural factors is likely to contribute to these differences [43]. In any case, this disparity must be taken into account by the operator during both the assessment and the ESWT session. In particular, the operator must increase the energy intensity very slowly when assessing women’s thresholds and scrupulously adhere to the thresholds found during the ESWT session, using an energy intensity that is intermediate between MiTI and MaTI.

Summarizing, the findings of the study demonstrate that the focused ESWT device can assess pain thresholds in musculoskeletal disorders but also identify painful joint sites and, consequently, damaged anatomical structures. They also suggest that the focused ESWT device can provide information on the severity of tissue damage and the course of musculoskeletal disorders and can assist the clinician in identifying musculoskeletal disorders and performing ESWT sessions.

These results can be achieved by using focused and not radial ESWT devices. Differently from radial shock waves, the focused shocks are concentrated on a limited area of the body and can penetrate deep into the tissue, whereas radial shock waves act with pressure at the skin’s surface and then diverge, although it cannot be excluded that they may also have a beneficial effect at depth [12].

Moreover, the transducer head of the focused shock wave device must not be held stationary at the site of maximum pain, but it must be moved over the entire joint surface, allowing a systematic exploration of the entire joint surface according to a specific procedure.

Currently, algometers are commonly used in clinical practice and clinical trials to measure the pain-pressure threshold in various tissues. Problematically, the pressure algometer measurements in the majority of these studies have been reported to be time-consuming, requiring laborious measurements to map sensitivity along a muscle or joint and ultimately construct a pressure sensitivity map, which is an appropriate assessment of pain in many clinical conditions [44].

Moreover, the results of pressure algometer measurement are influenced by different factors related to both the stimuli and the subjects, suggesting that the perception of pain induced by the 1-cm^2^ probe was sometimes difficult to define [45].

Unlike a pressure algometer, the focused ESWT device used according to the methodology we proposed allows large areas to be explored and pain to be mapped in a shorter time, and its results are less influenced by the method of mechanical stimulus delivery. For this reason, focused ESWT can be a valid alternative to the pressure algometer in clinical trials.

Despite these positive considerations, the study has several limitations.

The protocol we used required patients to report the moment they felt pain. This means that if the patient was not alert, he may have delayed reporting the onset of pain [32].

In addition, the device used in the study had a maximum mechanical energy level of 0.822 mJ/mm^2^, which created a “sky” effect. This may have led to an underestimation of MaTI and suggests that a device with a wider energy range should be used to study pain.

The study did not include a control group, but it compared data from injured joints with data from homologous healthy joints used as controls. This allowed us to assess pain without the interference of patients’ biopsychosocial factors, which could have influenced the results.

Instrumental examinations to assess the location and severity of tissue damage were not included in the study. The images may have confirmed that the device identifies damaged anatomical structures.

According to Leong et al. [20], the ESWT device may cause a blockade of mechanical nociceptors during treatment and affect the study results, but this should not have occurred in the study because the time needed to perform the ESWT assessment was usually quite short.

The study used a 6 Hz ESWT device and a 20 mm gel pad in all patients to standardize the assessments. It is unclear whether different frequencies and different gel pads would have yielded different results.

The study used digital pressure to locate and assess the pain. However, this method is difficult to quantify and standardize because of differences in the pressure applied by the same or different examiners and subjective reporting of pain by the patient [38].

Finally, the pain map used may have influenced the number of painful sites identified by the ESWT device.

## 5. Conclusions

Focused ESWT is a therapeutic tool, but when used appropriately, it can also identify damaged anatomical structures and provide information about the severity of tissue damage and the course of musculoskeletal disease.

For this reason, focused ESWT can be a valuable tool for clinicians when investigating pain and managing patients with musculoskeletal conditions.

## Figures and Tables

**Table 1:** Details of the areas belonging to the different joints that the physician has taken into account in the assessment of the sites of pain.

Joint	N. of Areas	Description of the Areas
Shoulder	5	Acromioclavicular area; supraspinatus pit; anterior side of the arm; lateral side of the arm; posterior side of the arm.
Elbow	3	Epicondylar region; epitrochlear region; forearm
Hip	4	Trochanteric area; peri trochanteric region; upper half of lateral side of the thigh; lower half of lateral side of the thigh.
Knee	5	Patellar area; peripatellar region; medial side of the knee; lateral side of the knee; rear side of the knee.
Foot	7	Heel; tarsus; forefoot; distal Achilles region; dorsal side of the foot; medial side of the foot; lateral side of the foot.

**Table 2:** Demographic and clinical characteristics of patients sample (*n* = 71).

Age, years	57.22 ± 11.30
Male/Female, *n*	17/54
Body Mass Index, kg/m^2^	26.26 ± 4.52
CIRS, severity score	0.74 ± 0.88
Depression or Anxiety: Yes/No	3/68
Diabetes: Yes/No	2/69
Previous ESWT execution: Yes/No	29/42
Time from onset to assessment, days	112.15 ± 6.83
Roles Maudsley score, at admission	3.42 ± 0.57
Roles Maudsley score, at 2 weeks	2.43 ± 0.83 *

Legend: Data are expressed as mean ± SD or number. CIRS = Cumulative Illness Rating Scale—Geriatrics; ESWT = extracorporeal shock wave therapy. Roles Maudsley score at admission vs. after 2 weeks: * *p* < 0.001.

**Table 3:** Profile of pain thresholds (mJ/mm^2^) assessed by ESWT at baseline, before the application of the first ESWT session.

Joints		Affected Joints		Healthy Joints	
	Number of Patients	MiTI	MaTI	MiTI	MaTI
Shoulder	31	0.214 ± 0.072	0.395 ± 0.136	0.262 ± 0.107	0.486 ± 0.162
Elbow	2	0.195 ± 0.060	0.369 ± 0.026	0.245 ± 0.035	0.486 ± 0.042
Hip	13	0.275 ± 0.153	0.506 ± 0236	0.369 ± 0.205	0.554 ± 0.224
Knee	1	0.113	0.27	0.138	0.351
Foot	24	0.200 ± 0.053	0.406 ± 0.153	0.269 ± 0.082	0.499 ± 0.171
ALL	71	0.218 ± 0.090 **	0.416 ± 0.165 *	0.282 ± 0.128	0.501 ± 0.174

Legend: Data are expressed as mean ± SD or number. MiTI: minimum threshold intensity; MaTI: maximum threshold intensity. * *p* = 0.003 referred to MaTI compared in the affected vs. health joints, and ** *p* < 0.001 referred to MiTI compared in the affected vs. health joints.

**Table 4:** Sites of pain elicited by digital pressure and ESWT evaluated in 71 patients, taking into account the patient’s highest subjective pain rating.

Joint		Pain Induced by Digital Pressure	Pain Induced by ESWT	
	Number of Patients	Number of Sites/ Max Number of Sites	Number of Sites/ Max Number of Sites	*p* Value
Shoulder	31	49/155	81/155	0.018
Elbow	2	4/6	4/6	1.000
Hip	13	27/52	51/52	0.038
Knee	1	2/5	3/5	0.714
Foot	24	41/168	65/168	0.041
ALL	71	123/386	204/386	0.0003

Legend: Data are expressed as numbers. ESWT= extracorporeal shock wave therapy. Comparisons were performed by chi-square test. A *p* < 0.05 was considered statistically significant.

## Data Availability

The original and anonymized data presented in the study will be made openly available at https:www.zenodo.org/ (accessed on 30 October 2024).

## References

[1] Haupt G. (1997). Use of extracorporeal shock waves in the treatment of pseudarthrosis, tendinopathy and other orthopedic diseases. J. Urol..

[2] Notarnicola A., Moretti B. (2012). The biological effects of extracorporeal shock wave therapy (ESWT) on tendon tissue. Muscles Ligaments Tendons J..

[3] Yalcin E., Keskin Akca A., Selcuk B., Kurtaran A., Akyuz M. (2012). Effects of extracorporal shock wave therapy on symptomatic heel spurs: A correlation between clinical outcome and radiologic changes. Rheumatol. Int..

[4] Simplicio C.L., Purita J., Murrell W., Santos G.S., Dos Santos R.G., Lana J.F.S.D. (2020). Extracorporeal shock wave therapy mechanisms in musculoskeletal regenerative medicine. J. Clin. Orthop. Trauma.

[5] Chao Y.H., Tsuang Y.H., Sun J.S., Chen L.T., Chiang Y.F., Wang C.C., Chen M.H. (2008). Effect of shock waves on tenocyte proliferation and extracellular matrix metabolism. Ultrasound Med. Biol..

[6] Wang C.J., Wang F.S., Yang K.D., Huang C.S., Hsu C.C. (2003). Shockwave therapy induced neovascularization at the tendon– bone junction. A study in rabbits. J. Orthop. Res..

[7] Wuerfel T., Schmitz C., Jokinen L.L.J. (2022). The Effects of the Exposure of Musculoskeletal Tissue to Extracorporeal Shock Waves. Biomedicines.

[8] Rompe J.D., Kirkpatrick C.J., Kullmer K., Schwitalle M., Krischnek O. (1998). Dose-related effects of shock waves on rabbit tendo Achillis: A sonographic and histological study. J. Bone Jt. Surg. Br..

[9] Lee S.Y., Cheng B., Grimmer-Somers K. (2011). The midterm effectiveness of extracorporeal shockwave therapy in the management of chronic calcific shoulder tendinitis. J. Shoulder Elbow Surg..

[10] Perwez A., Tiwari S., Madhual D., Prasad M., Sachan K. (2024). A Comprehensive Review of Extracorporeal Shockwave Therapy in the Management of Musculoskeletal Disorders: Efficacy, Mechanisms, and Clinical Applications. Int. J. Med. Sci. Clin. Res. Rev..

[11] Mishra B.N., Poudel R.R., Banskota B., Shrestha B.K., Banskota A.K. (2019). Effectiveness of extra-corporeal shock wave therapy (ESWT) vs methylprednisolone injections in plantar fasciitis. J. Clin. Orthop. Trauma.

[12] Pellegrino R., Di Iorio A., Filoni S., Mondardini P., Paolucci T., Sparvieri E., Tarantino D., Moretti A., Iolascon G. (2023). Radial or Focal Extracorporeal Shock Wave Therapy in Lateral Elbow Tendinopathy: A Real-Life Retrospective Study. Int. J. Environ. Res. Public Health.

[13] Furia J.P., Rompe J.D., Maffulli N. (2009). Low-energy extracorporeal shock wave therapy as a treatment for greater trochanteric pain syndrome. Am. J. Sports Med..

[14] Auersperg V., Trieb K. (2020). Extracorporeal shock wave therapy: An update. EFORT Open Rev..

[15] Tenforde A.S., Borgstrom H.E., DeLuca S., McCormack M., Singh M., Hoo J.S., Yun P.H. (2022). Best practices for extracorporeal shockwave therapy in musculoskeletal medicine: Clinical application and training consideration. PM R.

[16] Roerdink R.L., Dietvorst M., van der Zwaard B., van der Worp H., Zwerver J. (2017). Complications of extracorporeal shockwave therapy in plantar fasciitis: Systematic review. Int. J. Surg..

[17] Haake M., Böddeker I.R., Decker T., Buch M., Vogel M., Labek G., Maier M., Loew M., Maier-Boerries O., Fischer J. (2002). Side-effects of extracorporeal shock wave therapy (ESWT) in the treatment of tennis elbow. Arch. Orthop. Trauma Surg..

[18] Klonschinski T., Ament S.J., Schlereth T., Rompe J.D., Birklein F. (2011). Application of loca anesthesia inhibits effects of low-energy extracorporeal shock wave treatment (ESWT) on nociceptors. Pain Med..

[19] Rompe J.D., Furia J., Cacchio A., Schmitz C., Maffulli N. (2015). Radial shock wave treatment alone is less efficient than radial shock wave treatment combined with tissue-specific plantar fascia-stretching in patients with chronic plantar heel pain. Int. J. Surg..

[20] Leong H.T., Docking S., Girdwood M., Bonello C., Cook J., Rio E. (2019). Extracorporeal Shock Wave Therapy Immediately Affects Achilles Tendon Structure and Widespread Pressure Pain Thresholds in Healthy People: A Repeated-Measures Observational Study. Am. J. Phys. Med. Rehabil..

[21] Chow I.H., Cheing G.L. (2007). Comparison of different energy densities of extracorporeal shock wave therapy (ESWT) for the management of chronic heel pain. Clin. Rehabil..

[22] Lorusso L., Salerno M., Sessa F., Nicolosi D., Longhitano L., Loreto C., Carotenuto M., Messina A., Monda V., Villano I. (2018). Autoalgometry: An Important Tool for Pressure Pain Threshold Evaluation. J. Clin. Med..

[23] Tani K., Hirata A., Tanaka S. (2020). Quantitative Assessment of Pain Threshold Induced by a Single-Pulse Transcranial Magnetic Stimulation. Front. Neurosci..

[24] Giesbrecht R.J., Battié M.C. (2005). A comparison of pressure pain detection thresholds in people with chronic low back pain and volunteers without pain. Phys. Ther..

[25] de Goeij M., van Eijk L.T., Vanelderen P., Wilder-Smith O.H., Vissers K.C., van der Hoeven J.G., Kox M., Scheffer G.J., Pickkers P. (2013). Systemic inflammation decreases pain threshold in humans in vivo. PLoS ONE.

[26] Wu T., Li S., Ren J., Wang D., Ai Y. (2022). Efficacy of extracorporeal shock waves in the treatment of myofascial pain syndrome: A systematic review and meta-analysis of controlled clinical studies. Ann. Transl. Med..

[27] Konrad A., Kasahara K., Yoshida R., Murakami Y., Koizumi R., Nakamura M. (2023). Pain-Pressure Threshold Changes throughout Repeated Assessments with No Sex Related Differences. Healthcare.

[28] Kim E.S., Jo E.D., Han G.S. (2023). Effects of stretching intervention on musculoskeletal pain in dental professionals. J. Occup. Health.

[29] Salvi F., Miller M.D., Grilli A., Giorgi R., Towers A.L., Morichi V., Spazzafumo L., Mancinelli L., Espinosa E., Rappelli A. (2008). A manual of guidelines to score the modified cumulative illness rating scale and its validation in acute hospitalized elderly patients. J. Am. Geriatr. Soc..

[30] Ibrahim M.I., Donatelli R.A., Schmitz C., Hellman M.A., Buxbaum F. (2010). Chronic plantar fasciitis treated with two sessions of radial extracorporeal shock wave therapy. Foot Ankle Int..

[31] Leegaard A., Lomholt J.J., Thastum M., Herlin T. (2013). Decreased pain threshold in juvenile idiopathic arthritis: A cross-sectional study. J. Rheumatol..

[32] Vladimirova N., Jespersen A., Bartels E.M., Christensen A.W., Bliddal H., Danneskiold-Samsøe B. (2015). Pain Sensitisation in Women with Active Rheumatoid Arthritis: A Comparative Cross-Sectional Study. Arthritis.

[33] Jaber K., McAuliffe M., Pedler A., Sterling M., O’Leary S. (2022). Further exploring the relationship between pressure pain thresholds and function in knee osteoarthritis. Musculoskelet. Sci. Pract..

[34] Dainese P., Mahieu H., De Mits S., Wittoek R., Stautemas J., Calders P. (2023). Associations between markers of inflammation and altered pain perception mechanisms in people with knee osteoarthritis: A systematic review. RMD Open.

[35] Lohrer H., Nauck T., Korakakis V., Malliaropoulos N. (2016). Historical ESWT Paradigms Are Overcome: A Narrative Review. Biomed. Res. Int..

[36] Gleitz M., Hornig K. (2012). Triggerpunkte—Diagnose und Behandlungskonzepte unter besonderer Berücksichtigung extrakorporaler Stoßwellen [Trigger points—Diagnosis and treatment concepts with special reference to extracorporeal shockwaves]. Orthopade.

[37] Lou J., Wang S., Liu S., Xing G. (2017). Effectiveness of Extracorporeal Shock Wave Therapy Without Local Anesthesia in Patients with Recalcitrant Plantar Fasciitis: A Meta-Analysis of Randomized Controlled Trials. Am. J. Phys. Med. Rehabil..

[38] Chesterton L.S., Sim J., Wright C.C., Foster N.E. (2007). Interrater reliability of algometry in measuring pressure pain thresholds in healthy humans, using multiple raters. Clin. J. Pain.

[39] Moya D., Ramón S., Schaden W., Wang C.J., Guiloff L., Cheng J.H. (2018). The Role of Extracorporeal Shockwave Treatment in Musculoskeletal Disorders. J. Bone Jt. Surg. Am..

[40] Ryskalin L., Morucci G., Natale G., Soldani P., Gesi M. (2022). Molecular Mechanisms Underlying the Pain-Relieving Effects of Extracorporeal Shock Wave Therapy: A Focus on Fascia Nociceptors. Life.

[41] Maquet D., Croisier J.L., Demoulin C., Crielaard J.M. (2004). Pressure pain thresholds of tender point sites in patients with fibromyalgia and in healthy controls. Eur. J. Pain.

[42] Pelfort X., Torres-Claramunt R., Sánchez-Soler J.F., Hinarejos P., Leal-Blanquet J., Valverde D., Monllau J.C. (2015). Pressure algometry is a useful tool to quantify pain in the medial part of the knee: An intra- and inter-reliability study in healthy subjects. Orthop. Traumatol. Surg. Res..

[43] Pieretti S., Di Giannuario A., Di Giovannandrea R., Marzoli F., Piccaro G., Minosi P., Aloisi A.M. (2016). Gender differences in pain and its relief. Ann. Ist. Super. Sanita.

[44] Finocchietti S., Graven-Nielsen T., Arendt-Nielsen L. (2015). Dynamic mechanical assessment of muscle hyperalgesia in humans: The dynamic algometer. Pain Res. Manag..

[45] Duan G., Xiang G., Zhang X., Guo S., Zhang Y. (2014). An improvement of mechanical pain sensitivity measurement method: The smaller sized probes may detect heterogeneous sensory threshold in healthy male subjects. Pain Med..

